# Isolation of gene conferring salt tolerance from halophilic bacteria of Lunsu, Himachal Pradesh, India

**DOI:** 10.1186/s43141-020-00070-6

**Published:** 2020-10-06

**Authors:** Sonika Gupta, Parul Sharma, Kamal Dev, Anuradha Sourirajan

**Affiliations:** Faculty of Applied Sciences and Biotechnology, Shoolini University, Bajhol, PO Sultanpur, District Solan, Himachal Pradesh, 173229 India

**Keywords:** *Halobacillus trueperi*, Lunsu, Salt tolerance clone, Permease, Multidrug transport, Recombinant, pUC19, pGEX4T2

## Abstract

**Background:**

Halophiles offer an attractive source of genes conferring salt tolerance. *Halobacillus trueperi* SS1 strain of Lunsu, Himachal Pradesh, India, a strict halophile, was exploited to isolate and clone the genes for salt tolerance. The genomic library of BamH1 digest of *H. trueperi* SS1 was constructed in pUC19, and recombinants were screened for salt tolerance on an LB medium containing ampicillin (100 μg/ml) and NaCl (0 to 1.5 M).

**Results:**

One recombinant clone named as salt-tolerant clone (STC) conferred salt tolerance to host *Escherichia coli*/DH5α, which showed growth in the LB medium supplemented with ampicillin and 1.2 M NaCl. Restriction digestion and PCR analysis revealed the presence of an insert of approximately 2000 bp in the STC. DNA sequencing of the 2-kb insert on both strands yielded a sequence of 2301 nucleotides. Protein BLAST analysis of 2301-bp sequence of *H. trueperi* SS1 present in STC showed 97% identity to multidrug transport ATP binding/permease protein of *Halobacillus karajensis*. The insert contained in STC was subcloned into pGEX4T2 vector, and the recombinant clone STC/pGEX4T2 conferred salt tolerance to the bacterial host *E. coli*.

**Conclusions:**

The present study led to the isolation of salt tolerance gene encoding a putative multidrug transport ATP binding/permease protein from *H. trueperi* SS1. The salt tolerance gene can be subcloned for transferring salt tolerance traits into agricultural crop plants for cultivation in saline and coastal lands.

## Background

Earth is a salty planet, as most of its water is present in oceans and seas. This salinity feature has affected and continues to affect the land on which crops are or might be grown [1, 2]. Salinity is a major threat to food production and supply, because it limits crop yield and restricts the use of land [2].

Owing to their ability to survive in hyper-saline conditions, halophilic microbes serve as an attractive source for salt tolerance genes. Halophilic microorganisms have been explored for their application in agriculture, wherein conventional agricultural crops can be made more halotolerant by gene transfer from naturally halotolerant/halophilic microbes. Efforts are being made globally to isolate genes from microbes and plants and employ them for genetic engineering of salt tolerance in crops. Various genes responsible for providing salt tolerance and the ability to grow at elevated salt concentration have been identified from different bacteria [3], including the genes involved in synthesis and uptake of various compatible solutes [4]. These genes are important candidates for the generation of salt-tolerant recombinant microbial strains and transgenic plants [5, 6]. Two such salt tolerance genes were reported from a pond water metagenomic study, which encode for proteins with similarity to a putative general stress protein (GspM) harboring GsiB domain and a putative enoyl-CoA hydratase (EchM) [7]. To enhance salt tolerance in higher plants, numerous genes have been cloned, e.g., gene encoding late embryogenesis abundant proteins (*HVA*) [8], *P5CS* [9], *DREB1A* [10], and *AtNHX1* [11]. Alfin1 (a putative transcription factor) and *HAL1* (from yeast) have been engineered in alfalfa and tomato plants, respectively, to obtain salt-tolerant plants [12]. *HAL1* alters the salt tolerance of tomato and increases the K^+^/Na^+^ ratio in transgenic plants. Another choline oxidase gene from *Arthrobacter globiformis* has been transferred to *Arabidopsis thaliana* to enhance their salt tolerance [13]. The overexpression of fusion genes, *OsDST-SRDX* [14] and *AmRosea1* [15], significantly improved the salt tolerance of transgenic perennial ryegrass and rice, respectively. The isolation and identification of new salinity stress-induced genes will be useful in developing crop plants resistant to salinity stress. Previously, we reported the molecular characterization and halozyme production from halophiles of Lunsu salt water body of North-western Himalayas [16, 17]. In the present study, the strict halophile *Halobacillus trueperi* SS1 was explored for the genes conferring salt tolerance by selective screening of its genomic library.

## Methods

### Bacterial strain and genomic DNA isolation

Genomic DNA of *Halobacillus trueperi* SS1 (Genbank Accession no. KM260166) was isolated as described [16]. *Halobacillus trueperi* SS1 was isolated from Lunsu soil sample as described [16]. *H*. *trueperi* SS1 is a strict halophile and exhibits growth up to 4.5 M NaCl with the optimal growth at 2 M NaCl.

### Genomic library construction

Plasmid DNA isolation, restriction enzyme digestion, ligation, and competent cell preparation were carried out by standard procedures [18]. Ten micrograms of *H. trueperi* SS1 genomic DNA was partially digested with BamH1, and DNA fragments in the range of 1–6 kb were excised from agarose gel (0.8%) using gel extraction kit (Fermentas Inc. USA). Two hundred nanograms of partially digested DNA was ligated to BamHI digested pUC19 vector by using T4 DNA ligase. The ligated product was transformed into *E. coli* DH5α competent cells, and transformants were plated on LB agar plates containing ampicillin (100 μg/ml) and X-Gal/IPTG for blue-white selection.

### Screening of recombinant genomic clones of *H. trueperi* SS1 for salt tolerance

For screening of salt tolerance gene(s), *E. coli* transformants harboring genomic DNA clones of *H. trueperi* SS1 genes in pUC19 were plated on LB ampicillin media containing 0.75 M NaCl. Such a selection allowed the growth of only those *E. coli* transformants that contain gene(s) for salt tolerance. The transformants that showed growth in the presence of NaCl were selected and used for the isolation of plasmid by alkaline lysis [18]. The plasmid DNA was analyzed by restriction digestion for the release of insert.

### Functional characterization of genomic clones of *H. trueperi* SS1 conferring salt tolerance to *E. coli*/DH5α

The growth pattern of the genomic clone of *H. trueperi* SS1 was studied in the LB agar medium and LB agar medium supplemented with ampicillin and different concentrations of NaCl (0.5, 0.75, 1, 1.2, and 1.5 M). The *E. coli*/DH5α harboring genomic clone and control *E. coli*/pUC19 were cultured in the LB medium supplemented with ampicillin, while *E. coli*/DH5α in LB alone, by incubating at 37 °C for 24 h with shaking at 200 rpm. The absorbance of the culture was measured at 600 nm, and cell density was normalized to *A*_600_ = 1. Serial dilutions of equal number of cells of each strain were spotted on different media as described, and the plates were incubated at 37 °C for 24 h.

The salt tolerance of the genomic clone was further analyzed by liquid growth assay in the LB medium supplemented with ampicillin and different concentrations of NaCl (0, 0.75, and 1.0 M NaCl). Incubation was done at 37 °C for 24 h with shaking at 200 rpm. The absorbance of the culture was measured at 600 nm at various time intervals and the growth curve was plotted.

### Confirmation of recombinant genomic clone

The plasmid DNA of salt-tolerant clone (STC) was used as a template for PCR amplification by using pUC19-specific primers (pUC19 F 5′ GTAAAACGACGGCCAGT 3′ and pUC19 R 5′ CAGGAAACAGCTATGAC 3′). PCR mixture was prepared by mixing the components (1 μM of each primer, 0.2 mM each of dNTPs, 100 ng of plasmid DNA, and 1 U of Taq DNA polymerase). The reaction mixture tubes were subjected for thermal cycling reaction of initial denaturation at 94 °C for 2 min, followed by 35 cycles of denaturation (94 °C, 30 s), annealing (40 °C, 30 s), and extension (72 °C, 2 min), with a final extension of 10 min at 72 °C. The results were analyzed using 1.2% agarose gel and stained with ethidium bromide and visualized with an UV gel documentation system.

For verification of the recombinant clone for salt tolerance traits, it was subcloned into pGEX4T2 expression vector by using appropriate restriction enzymes and analyzed for salt tolerance.

### Identification of genes conferring salt tolerance in the recombinant genomic clone

The gene conferring salt tolerance was subjected to DNA sequencing on both strands using pUC19-specific forward and reverse primers. The plasmid DNA sequencing was carried out at Eurofins-Operon Pvt Ltd, Bangalore, India. DNA sequence was assembled and translated to amino acid sequence using Expasy tools. The Translate tool namely ExPASy Protein BLAST was used for multiple alignment of sequence, and the open reading frame (ORF) was predicted using an ORF finder tool at NCBI (http://www.ncbi.nlm.nih.gov/gorf/gorf.html) and analyzed for the database homology with BLAST (http://www.ncbi.nlm.nih.gov/blast).

## Results and discussion

### Isolation of gene(s) conferring salt tolerance from *H. trueperi* SS1

**Fig. 1:**
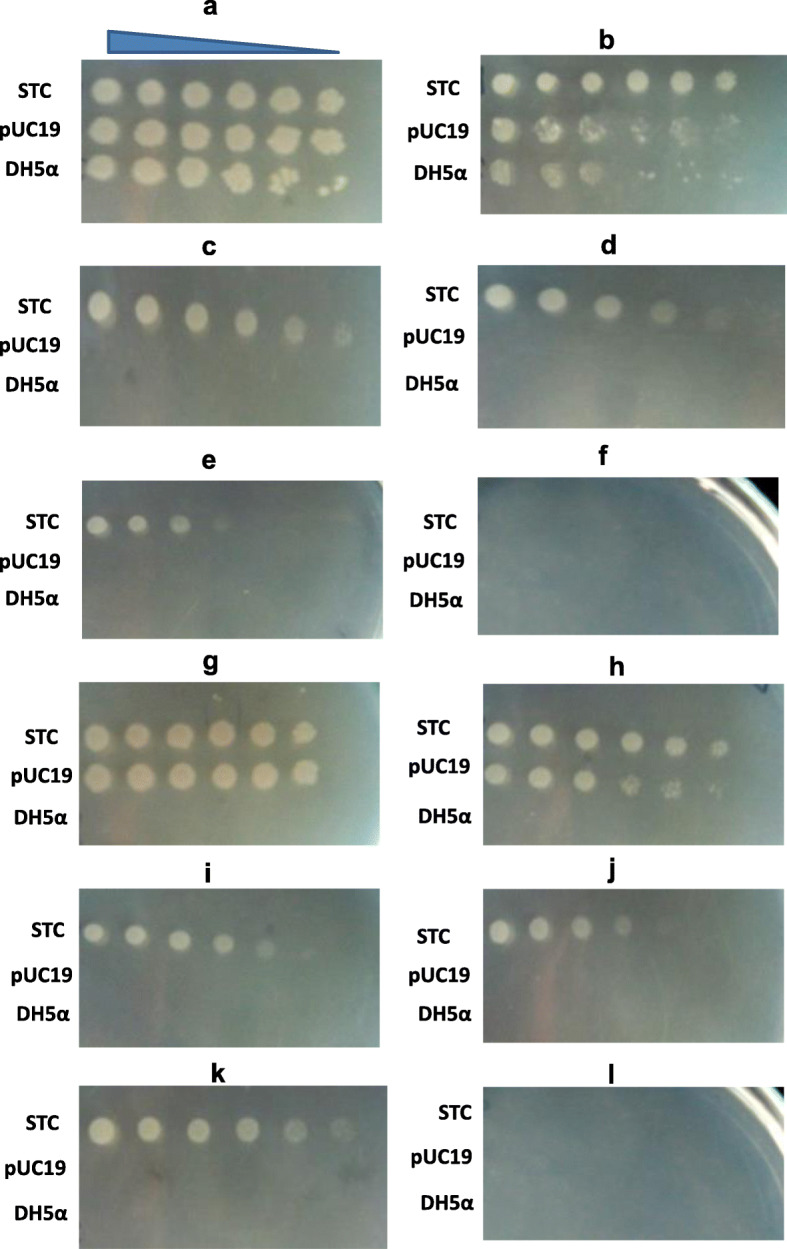
Growth characteristics of recombinant clone STC. The growth of recombinant clone STC, DH5α transformed with pUC19, and DH5α host strain were analyzed for NaCl tolerance in the LB agar medium. A tenfold serial dilution (from left to right as indicated above panel **a**) of each strain was spotted on the LB medium (**a**–**f**) and LB medium containing ampicillin (**g**–**l**) and increasing concentration of NaCl as follows: 0 (**a**, **g**), 0.5 (**b**, **h**), 0.75 (**c**, **i**), 1.0 (**d**, **j**), 1.2 (**e**, **k**) and 1.5 M (**f**, **l**). The medium without NaCl was used as control

Owing to the ability of halophilic isolate *H. trueperi* SS1 to survive in hyper-saline conditions (4.5 M NaCl), it was used to isolate potential salt tolerance genes. Since there is limited information on the genome of extremophiles like *H. trueperi*, the study was carried out to generate a genomic library and used to screen for genes/gene clusters for salt tolerance. To isolate genes conferring salt tolerance, a genomic library was constructed from *BamH1* digested *H. trueperi* SS1 DNA. For the construction of the genomic library of *H. trueperi* SS1, pUC19 cloning vector was selected owing to its potential of blue/white recombinant screening. Blue/white screening identified > 200 white colonies. All the recombinant clones were screened for salt (NaCl) tolerance in a growth medium supplemented with 0.75 M NaCl and ampicillin. A recombinant clone designated as salt-tolerant clone (STC) showed growth on the medium supplemented with 0.75 M NaCl and ampicillin, after incubation at 37 °C for 24 h, indicating salt-tolerant nature of the transformant (Fig. 1).

The isolation of salt-tolerant clones has been reported from pond water metagenome which showed growth in the medium containing 750 mM NaCl, in comparison to *E. coli*/pUC19 [7].

Similarly, Ahmed et al. [19] reported recombinant plasmids named as *pSSR1*, *pSSR4*, *pSSR6*, and *pSSR21*, which showed significant growth in the presence of NaCl [4.0% (w/v)] and KCl [5.5% (w/v)] as compared to *E. coli* (DH10B) strain carrying empty plasmid vector (*pUC*19) [19].

### Characteristics of salt tolerance of recombinant clone STC

The growth of recombinant clone STC was checked by spotting assay on the LB agar medium containing 0.5, 0.75, 1.0, 1.2, and 1.5 M NaCl (Fig. 1a–f) and the LB agar medium supplemented with ampicillin and different concentrations of NaCl (0–1.5 M) (Fig. 1g–l). Interestingly, recombinant clone STC showed detectable growth up to 1.2 M NaCl, where as host strain *E. coli* DH5α and DH5α transformed with pUC19 (vector alone) did not show any growth above 0.5 M NaCl. These results clearly support the fact that NaCl tolerance is an intrinsic property and STC clone might possess gene(s) that confer salt tolerance to *E. coli* strain DH5α.

In another study, the RelA/SpoT homolog Sj-RSH was isolated from *Suaeda japonica* cDNA library [20]. The Sj-RSH transformant grew in the presence of 0.4 M NaCl as compared to other transformants, which showed growth in the presence of 0.086 M NaCl. It was concluded that gene encoding Sj-RSH might play an important role in the salt- and osmotic-stress tolerance conferred to *E. coli* [20]. On the other hand, the STC clone, isolated in the present study, conferred salt tolerance up to 1.2 M NaCl.

**Fig. 2:**
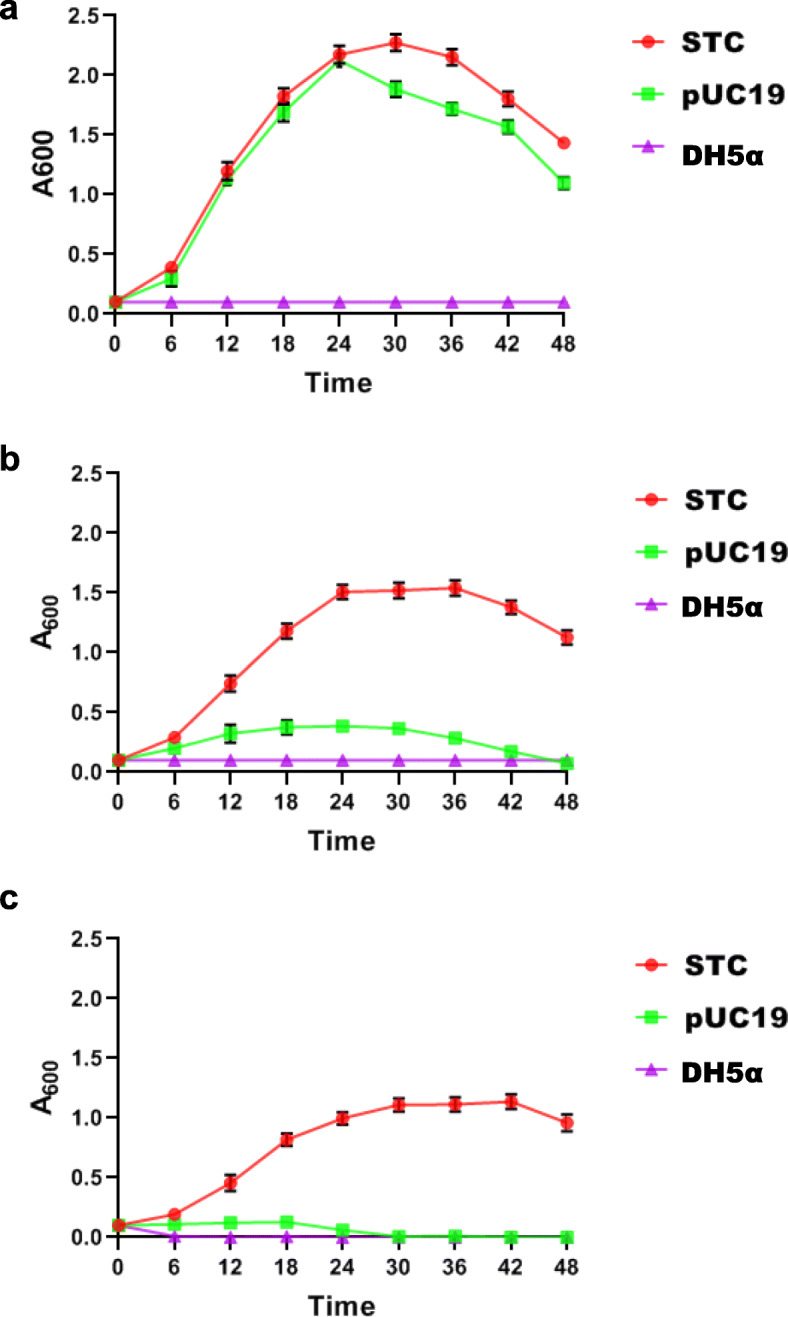
Growth kinetics of recombinant clone STC. Panel **a** represents control with ampicillin and without added salt; panels **b** and **c** represent salinity treatment with 0.75 M NaCl and 1 M NaCl, respectively, in the presence of ampicillin. Recombinant clone STC (red line) in DH5α, DH5α containing pUC19 (green line), and DH5α host strain (violet line) were inoculated in the LB medium supplemented with ampicillin (**a**), LB medium supplemented with ampicillin and 0.75 M NaCl (**b**), and LB medium supplemented with ampicillin and 1 M NaCl (**c**). Cells were withdrawn at different intervals, and absorbance was measured at 600 nm. Data of three independent experiments was plotted with standard deviation

The salt tolerance of DH5α/STC was characterized by analysis of the growth kinetics in liquid media as follows: LB medium (Fig. 2a), LB medium supplemented with ampicillin (100 μg/ml) and 0.75 M NaCl (Fig. 2b), and LB medium supplemented with ampicillin (100 μg/ml) and 1 M NaCl (Fig. 2c). The recombinant strain *E. coli/*STC exhibited a hyperbolic growth pattern and achieved maximum growth in 24–28 h in the medium containing 0.75 and 1 M NaCl (Fig. 2b, c). On the other hand, the control *E. coli/*pUC19 and *E. coli/*DH5α did not show any significant growth in the presence of salt (Fig. 2b, c). These results reinforce the fact that the STC clone confers salt tolerance to the *E. coli* strain harboring the recombinant clone.

### Verification of the STC clone

To verify and determine the size of the insert in the STC, the recombinant plasmid DNA of the STC clone was digested with *BamH1*. *BamH1* digestion released a DNA fragment of ~ 2 kb (Supplementary Fig S1a), indicating the presence of an insert ~ 2 kb. The presence of the insert was also confirmed by PCR analysis using pUC19 primers flanking the MCS. An amplicon of ~ 2 kb was amplified from STC, which confirms restriction analysis (Supplementary Fig S1b).

### Identification of STC

To identify the genes present in the insert in STC, the insert was sequenced on both strands of DNA. A nucleotide sequence of 2301 bp was obtained (Supplementary Fig S2) and analyzed by BLAST. The DNA sequence was translated into six frames by using Expasy tool, and each frame was subjected to protein BLAST (Table 1).

**Table 1:** Protein BLAST analysis of the *H. trueperi* SS1 genomic DNA insert present in STC. The predicted ORFs and their BLAST hits are indicated

Clone STC	Open reading frame (ORF)	Best match			
		Protein name accession number	Size aa	Organism	Identity
**Insert size** 2301 bp	3′–5′ FRAME 3	Putative multidrug export ATP binding/permease protein	101	*Halobacillus karajensis* GN=BN983_ 02429	95/98 = 97%
	3′–5′ FRAME 2	Putative multidrug export ATP binding/permease protein	153	*Halobacillus karajensis* GN=BN983 _02429	134/143 = 94%
	5′–3′ FRAME 2	Putative membrane protein	165	*Halobacillus karajensis* GN=BN983 _02430	148/165 = 90%
	3′–5′ FRAME 1	Multidrug ABC transporter ATP binding protein OS	99	*Halobacillus* sp. *BBL2006*	23/34 = 64%
	5′–3′ FRAME 3	Membrane protein	49	*Aquimarina* sp. *GN=AT012 _12720*	13/39 = 33%
	5′–3′ FRAME 1	Glycosyl transferase family	29	*Geobacter lovleyi* *ATCC BAA-1151*	16/55 = 29%

Out of the six open reading frames (ORFs), two frames gave the highest identity (97 and 94%, respectively) with Putative multidrug export ATP binding/permease protein, and the third frame gave 90% identity with putative membrane protein from *Halobacillus* sp. (Table 1). The ATP-binding cassette transporters (ABC transporters) are a transport system superfamily. These genes encode integral membrane proteins that translocate a wide range of solutes across membranes [21]. ABC proteins can act as importers, exporters, receptors, and channels. ABC uptake porters take up a large variety of nutrients, biosynthetic precursors, trace metals, and vitamins, while exporters transport lipids, sterols, drugs, and a large variety of primary and secondary metabolites [22].

Ahmed et al. [19] reported a putative branched-chain amino acid (BCAA) ABC transporter gene (*BCAA_ABCTP*) (putative *BCAA_ABCTP*) gene, which was subcloned (*pSSR1C1*) in salt-sensitive *E. coli* strain MKH13. The transformed strain bearing *pSSR1C1* showed a significant growth in the presence of NaCl [3.0% (w/v)] and KCl [3.7% (w/v)] as compared to the strain carrying only the empty vector (*pUC*19) [19]. In another study, a metagenomic library from the human gut microbiota was screened for potential salt-tolerant clones [23]. Transformed clones showed a significant increase in salt tolerance (0.5 M NaCl) compared with the wild-type *E. coli* strain MKH13. Five genes were identified in the clone namely galE (3), galE (25), mazG (3), mazG (5), and murB (3) [23].

### Verification of salt tolerance trait of the STC by retransformation

To further confirm the presence of the salt tolerance gene in the 2-kb insert of STC, the insert was released and subcloned into pGEX 4T2 vector. The vector pGEX4T2 was selected towards subcloning and reconfirmation of salt tolerance of the genomic insert of the STC clone. The transformants were checked for growth on media supplemented with NaCl (0–1.5 M). The transformants containing pGEX4T2/STC grew on the media supplemented with ampicillin and 0–1.0 M NaCl (Supplementary Fig S3a-j), while the control strain containing pGEX4T2 vector failed to grow on the media supplemented with 0.75 M NaCl (Supplementary Fig S3). These results indicate the plasmid-borne nature of salt tolerance (pGEX4T2/STC). Restriction digestion of the clone pGEX4T2/STC revealed that it contained an insert of ~ 2.0 kb (data not shown).

Similarly, *Agrobacteium tumifaciens* strain LBA 4404 was transformed with the gene encoding choline oxidase [24]. The transformants were able to survive in the presence of 0.3 M NaCl. Deshnium et al. [24] also observed increased salinity tolerance up to 0.4 M NaCl in *Synechococus* sp. when transformed with *codA* gene encoding choline oxidase.

In the present study, the *E. coli* strain DH5α by itself exhibited growth in the presence of 0.5 M NaCl. Accordingly, the Sj-RSH clone identified by Yamada et al. [20] might not qualify to be *a* salt tolerance clone. The comparison of salt tolerance of STC isolated in this study with those reported in literature reveals that STC confers the highest salt tolerance (up to 1.2 M NaCl) to its host strain (*E. coli*) amongst all the salt-tolerant genes reported so far.

Sequencing of the genomic DNA insert of *H. trueperi* SS1 in STC indicated the presence of gene homologous to putative multidrug export ATP binding/permease of *Halobacillus karajensis* (Table 1). Consistent with these results, genome sequencing of *Halobacillus trueperi* SS1 revealed the presence of permeases/transporters [25]. Further analysis of the genome sequence will shed light into the mapping of the salt tolerance gene isolated in this study. Studies on the mechanism of salt tolerance in *H. trueperi* SS1 showed that the strict halophile utilizes a combination of salt-in strategy and accumulation of compatible solutes to combat salt stress [26]. Since both strategies require transport proteins, the putative permease homolog of *Halobacillus karajensis* identified in the STC clone might be involved in Na^+^ and K^+^ transport or in the accumulation of organic osmolytes (proline, glycine, sugars, or alcohols) to confer salt-tolerant growth to native *Halobacillus trueperi* SS1 and to the transformed *E. coli* host.

## Conclusion

The present study led to the isolation of gene conferring salt tolerance (up to 1.2 M NaCl) from the genomic library of *H. trueperi* SS1. Further studies on cloning and biochemical characterization of the putative permease of *H. trueperi* SS1 will shed light into the mechanism of salt tolerance. The salt tolerance gene (putative multidrug export ATP binding/permease protein) isolated from *H. trueperi* SS1 can serve as a tool for engineering salt tolerance in agricultural crop plants like rice, the major crop grown in coastal regions suffering salinity stress.

## Supplementary information

**Additional file 1: Figure S1.** Restriction digestion and PCR analysis of STC clone. (a) Restriction digestion of pUC19 (vector) and STC (recombinant clone) with *BamH1*. Lane1. pUC19 plasmid DNA; lane 2, pUC19 plasmid DNA digested with *BamH1*; Lane 3, plasmid DNA of clone STC and lane 4, plasmid DNA of STC digested with *BamH1*. (b) PCR analysis of STC with pUC19 primers flanking multiple cloning site. Lane 1, PCR amplified product of pUC19; and Lane 2, PCR amplified product of clone STC. Lane M indicates the DNA molecular size marker (kb). The release of an insert of ~2 kb and its specific PCR amplification are indicated. **Figure S2.** Nucleotide sequence of *H. trueperi* SS1 genomic DNA insert in STC clone corresponding to 2301 bp. The insert was sequenced on both strands using pUC19 primers. The two sequences were aligned and overlaps were removed to assemble the sequence of 2301 bp. **Figure S3.** Growth characteristics of STC subcloned in pGEX4T2. The growth of recombinant clone STC, control DH5α transformed with pGEX4T2 and DH5α host strain were analyzed for NaCl tolerance in LB agar medium. A ten fold serial dilution (from left to right as indicated above panel a) of each strain was spotted on LB medium (a - e) and LB medium containing ampicillin (f – j) and increasing concentration of NaCl as follows: 0 (a and f), 0.5 (b and g), 0.75 (c and h), 1.0 (d and i) and 1.5 M (e and j).

## Data Availability

Additional data is provided as supplementary material.

## Abbreviations

BLAST: Basic local alignment search tool
DNA: Deoxyribonucleic acid
*E. coli*: *Escherichia coli*
IPTG: Isopropyl β-d-1-thiogalactopyranoside
LB: Luria-Bertani
MCS: Multiple cloning site
NaCl: Sodium chloride
UV: Ultraviolet
X-gal: 5-Bromo-4-chloro-3-indolyl-β-d-galactopyranoside
