# Supplementary material for: Isolation of gene conferring salt tolerance from halophilic bacteria of Lunsu, Himachal Pradesh, India
Source: J Genet Eng Biotechnol. 2020 Oct 6;18:57. doi: 10.1186/s43141-020-00070-6 (PMC7538504; doi:10.1186/s43141-020-00070-6)
Supplement: Supplementary file 1 — Additional file 1: Figure S1. Restriction digestion and PCR analysis of STC clone. (a) Restriction digestion of pUC19 (vector) and STC (recombinant clone) with BamH1. Lane1. pUC19 plasmid DNA; lane 2, pUC19 plasmid DNA digested with BamH1; Lane 3, plasmid DNA of clone STC and lane 4, plasmid DNA of STC digested with BamH1. (b) PCR analysis of STC with pUC19 primers flanking multiple cloning site. Lane 1, PCR amplified product of pUC19; and Lane 2, PCR amplified product of clone STC. Lane M indicates the DNA molecular size marker (kb). The release of an insert of ~2 kb and its specific PCR amplification are indicated. Figure S2. Nucleotide sequence of H. trueperi SS1 genomic DNA insert in STC clone corresponding to 2301 bp. The insert was sequenced on both strands using pUC19 primers. The two sequences were aligned and overlaps were removed to assemble the sequence of 2301 bp. Figure S3. Growth characteristics of STC subcloned in pGEX4T2. The growth of recombinant clone STC, control DH5α transformed with pGEX4T2 and DH5α host strain were analyzed for NaCl tolerance in LB agar medium. A ten fold serial dilution (from left to right as indicated above panel a) of each strain was spotted on LB medium (a - e) and LB medium containing ampicillin (f – j) and increasing concentration of NaCl as follows: 0 (a and f), 0.5 (b and g), 0.75 (c and h), 1.0 (d and i) and 1.5 M (e and j). [file 43141_2020_70_MOESM1_ESM.pptx]

## Slide 1
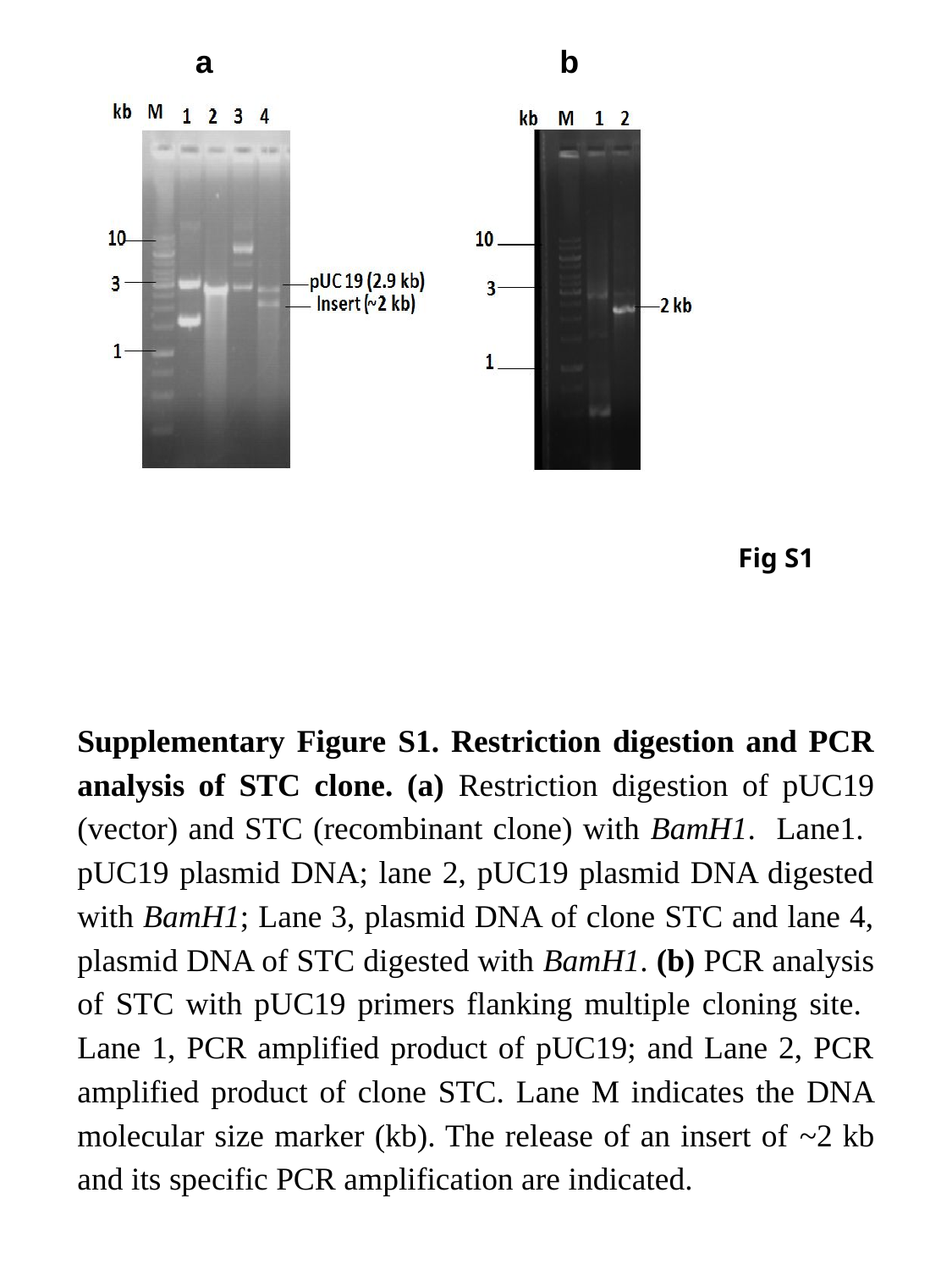

a
b
Fig S1
Supplementary Figure S1. Restriction digestion and PCR analysis of STC clone. (a) Restriction digestion of pUC19 (vector) and STC (recombinant clone) with BamH1. Lane1. pUC19 plasmid DNA; lane 2, pUC19 plasmid DNA digested with BamH1; Lane 3, plasmid DNA of clone STC and lane 4, plasmid DNA of STC digested with BamH1. (b) PCR analysis of STC with pUC19 primers flanking multiple cloning site. Lane 1, PCR amplified product of pUC19; and Lane 2, PCR amplified product of clone STC. Lane M indicates the DNA molecular size marker (kb). The release of an insert of ~2 kb and its specific PCR amplification are indicated.

## Slide 2
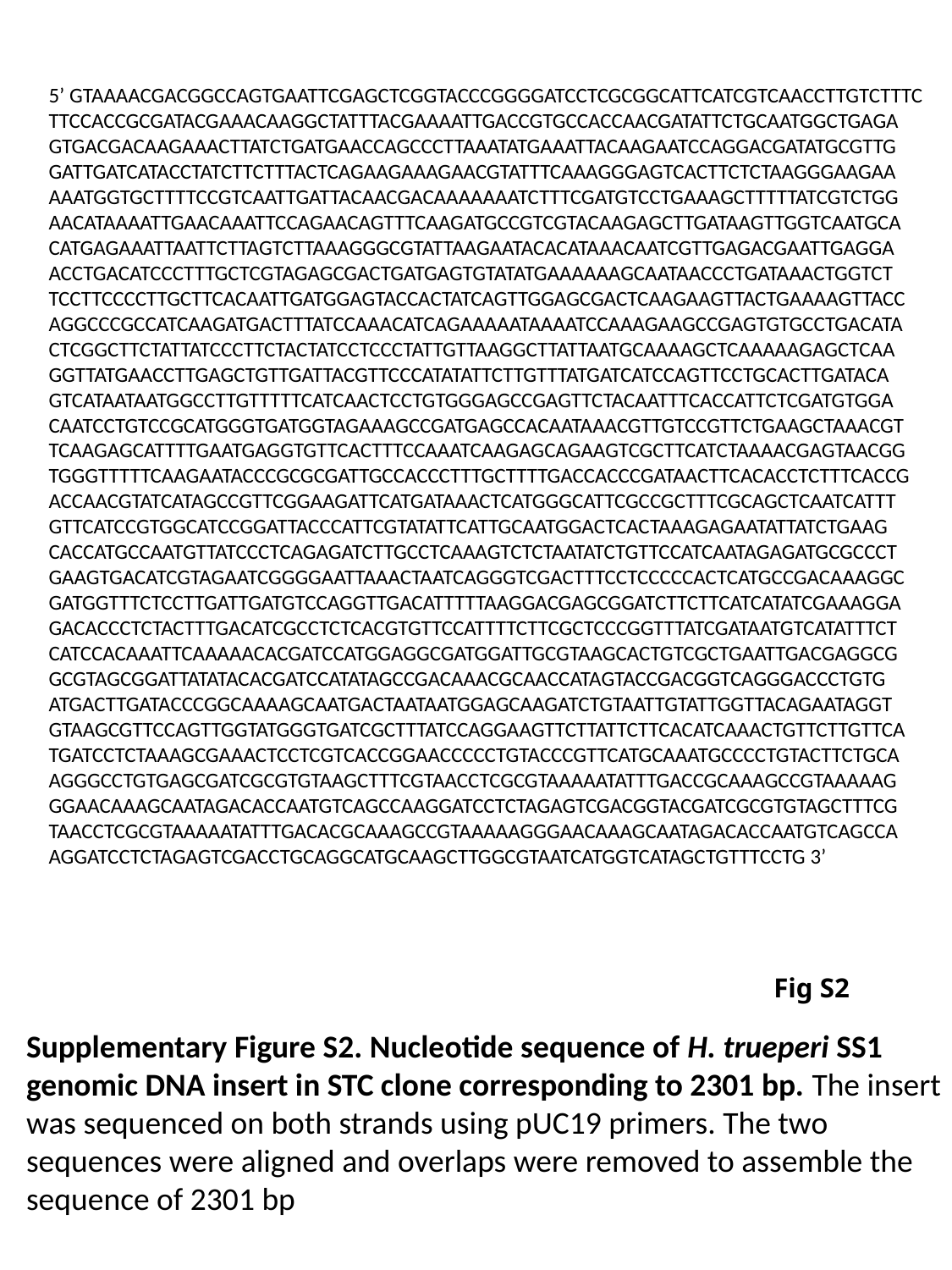

5’ GTAAAACGACGGCCAGTGAATTCGAGCTCGGTACCCGGGGATCCTCGCGGCATTCATCGTCAACCTTGTCTTTC
TTCCACCGCGATACGAAACAAGGCTATTTACGAAAATTGACCGTGCCACCAACGATATTCTGCAATGGCTGAGA
GTGACGACAAGAAACTTATCTGATGAACCAGCCCTTAAATATGAAATTACAAGAATCCAGGACGATATGCGTTG
GATTGATCATACCTATCTTCTTTACTCAGAAGAAAGAACGTATTTCAAAGGGAGTCACTTCTCTAAGGGAAGAA
AAATGGTGCTTTTCCGTCAATTGATTACAACGACAAAAAAATCTTTCGATGTCCTGAAAGCTTTTTATCGTCTGG
AACATAAAATTGAACAAATTCCAGAACAGTTTCAAGATGCCGTCGTACAAGAGCTTGATAAGTTGGTCAATGCA
CATGAGAAATTAATTCTTAGTCTTAAAGGGCGTATTAAGAATACACATAAACAATCGTTGAGACGAATTGAGGA
ACCTGACATCCCTTTGCTCGTAGAGCGACTGATGAGTGTATATGAAAAAAGCAATAACCCTGATAAACTGGTCT
TCCTTCCCCTTGCTTCACAATTGATGGAGTACCACTATCAGTTGGAGCGACTCAAGAAGTTACTGAAAAGTTACC
AGGCCCGCCATCAAGATGACTTTATCCAAACATCAGAAAAATAAAATCCAAAGAAGCCGAGTGTGCCTGACATA
CTCGGCTTCTATTATCCCTTCTACTATCCTCCCTATTGTTAAGGCTTATTAATGCAAAAGCTCAAAAAGAGCTCAA
GGTTATGAACCTTGAGCTGTTGATTACGTTCCCATATATTCTTGTTTATGATCATCCAGTTCCTGCACTTGATACA
GTCATAATAATGGCCTTGTTTTTCATCAACTCCTGTGGGAGCCGAGTTCTACAATTTCACCATTCTCGATGTGGA
CAATCCTGTCCGCATGGGTGATGGTAGAAAGCCGATGAGCCACAATAAACGTTGTCCGTTCTGAAGCTAAACGT
TCAAGAGCATTTTGAATGAGGTGTTCACTTTCCAAATCAAGAGCAGAAGTCGCTTCATCTAAAACGAGTAACGG
TGGGTTTTTCAAGAATACCCGCGCGATTGCCACCCTTTGCTTTTGACCACCCGATAACTTCACACCTCTTTCACCG
ACCAACGTATCATAGCCGTTCGGAAGATTCATGATAAACTCATGGGCATTCGCCGCTTTCGCAGCTCAATCATTT
GTTCATCCGTGGCATCCGGATTACCCATTCGTATATTCATTGCAATGGACTCACTAAAGAGAATATTATCTGAAG
CACCATGCCAATGTTATCCCTCAGAGATCTTGCCTCAAAGTCTCTAATATCTGTTCCATCAATAGAGATGCGCCCT
GAAGTGACATCGTAGAATCGGGGAATTAAACTAATCAGGGTCGACTTTCCTCCCCCACTCATGCCGACAAAGGC
GATGGTTTCTCCTTGATTGATGTCCAGGTTGACATTTTTAAGGACGAGCGGATCTTCTTCATCATATCGAAAGGA
GACACCCTCTACTTTGACATCGCCTCTCACGTGTTCCATTTTCTTCGCTCCCGGTTTATCGATAATGTCATATTTCT
CATCCACAAATTCAAAAACACGATCCATGGAGGCGATGGATTGCGTAAGCACTGTCGCTGAATTGACGAGGCG
GCGTAGCGGATTATATACACGATCCATATAGCCGACAAACGCAACCATAGTACCGACGGTCAGGGACCCTGTG
ATGACTTGATACCCGGCAAAAGCAATGACTAATAATGGAGCAAGATCTGTAATTGTATTGGTTACAGAATAGGT
GTAAGCGTTCCAGTTGGTATGGGTGATCGCTTTATCCAGGAAGTTCTTATTCTTCACATCAAACTGTTCTTGTTCA
TGATCCTCTAAAGCGAAACTCCTCGTCACCGGAACCCCCTGTACCCGTTCATGCAAATGCCCCTGTACTTCTGCA
AGGGCCTGTGAGCGATCGCGTGTAAGCTTTCGTAACCTCGCGTAAAAATATTTGACCGCAAAGCCGTAAAAAG
GGAACAAAGCAATAGACACCAATGTCAGCCAAGGATCCTCTAGAGTCGACGGTACGATCGCGTGTAGCTTTCG
TAACCTCGCGTAAAAATATTTGACACGCAAAGCCGTAAAAAGGGAACAAAGCAATAGACACCAATGTCAGCCA
AGGATCCTCTAGAGTCGACCTGCAGGCATGCAAGCTTGGCGTAATCATGGTCATAGCTGTTTCCTG 3’
Fig S2
Supplementary Figure S2. Nucleotide sequence of H. trueperi SS1 genomic DNA insert in STC clone corresponding to 2301 bp. The insert was sequenced on both strands using pUC19 primers. The two sequences were aligned and overlaps were removed to assemble the sequence of 2301 bp

## Slide 3
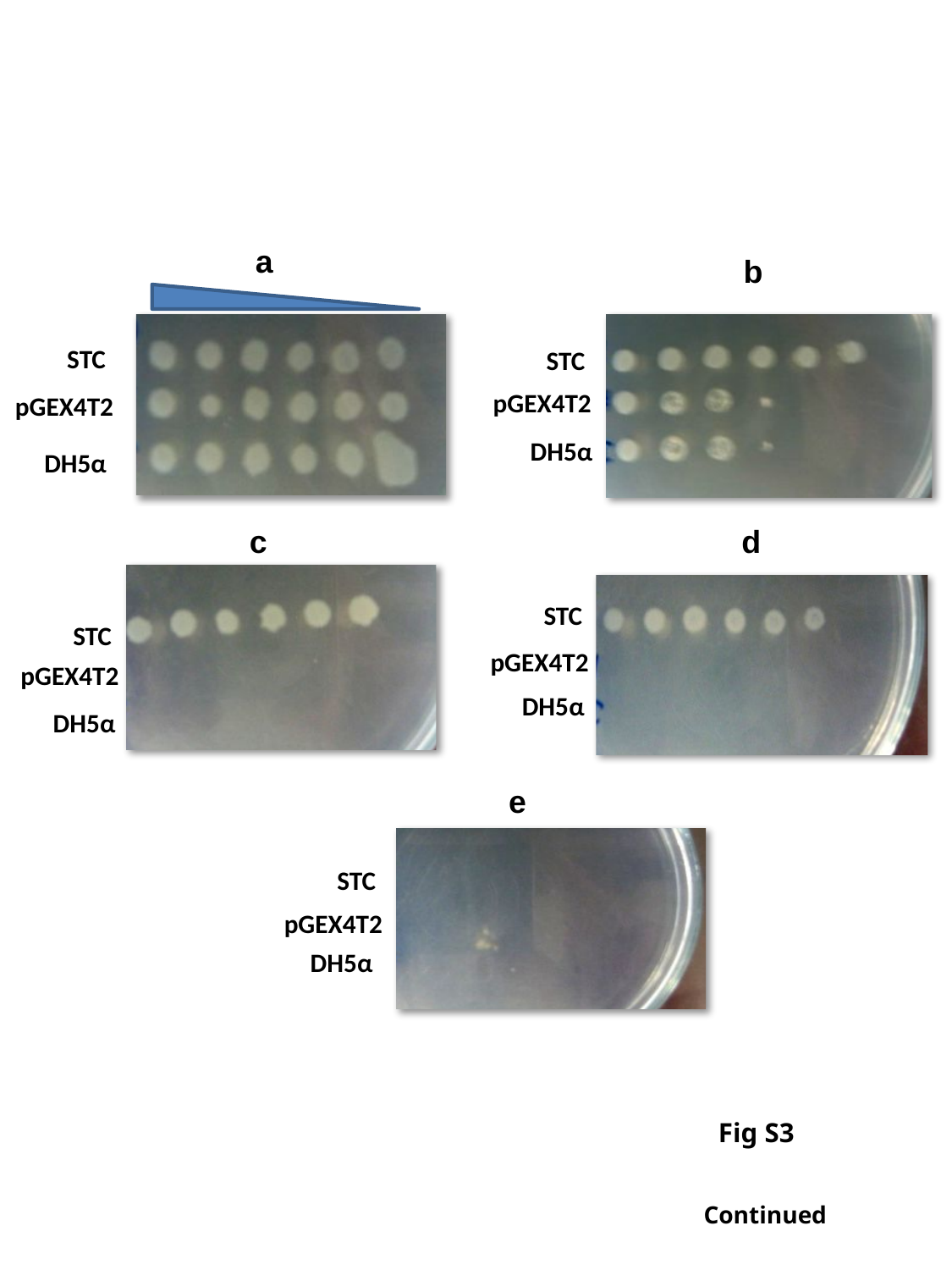

a
b
STC
STC
pGEX4T2
pGEX4T2
DH5α
DH5α
c
d
STC
STC
pGEX4T2
pGEX4T2
DH5α
DH5α
e
STC
pGEX4T2
DH5α
Fig S3
Continued

## Slide 4
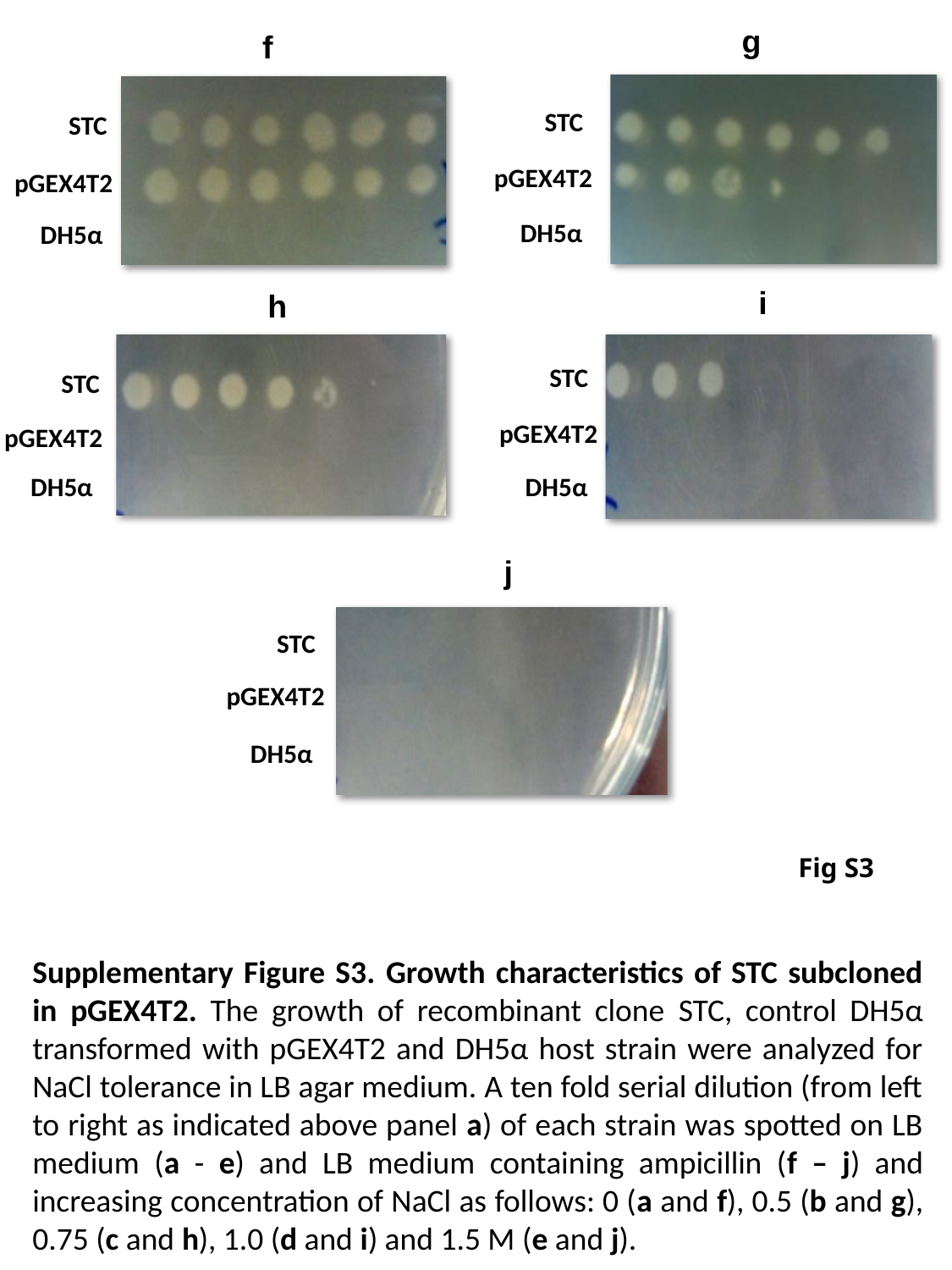

g
f
STC
STC
pGEX4T2
pGEX4T2
DH5α
DH5α
i
h
STC
STC
pGEX4T2
pGEX4T2
DH5α
DH5α
j
STC
pGEX4T2
DH5α
Fig S3
Supplementary Figure S3. Growth characteristics of STC subcloned in pGEX4T2. The growth of recombinant clone STC, control DH5α transformed with pGEX4T2 and DH5α host strain were analyzed for NaCl tolerance in LB agar medium. A ten fold serial dilution (from left to right as indicated above panel a) of each strain was spotted on LB medium (a - e) and LB medium containing ampicillin (f – j) and increasing concentration of NaCl as follows: 0 (a and f), 0.5 (b and g), 0.75 (c and h), 1.0 (d and i) and 1.5 M (e and j).
